# The Regulation of CD4^+^ T Cell Responses during Protozoan Infections

**DOI:** 10.3389/fimmu.2014.00498

**Published:** 2014-10-13

**Authors:** Christian R. Engwerda, Susanna S. Ng, Patrick T. Bunn

**Affiliations:** ^1^QIMR Berghofer Medical Research Institute, Brisbane, QLD, Australia; ^2^School of Natural Sciences, Griffith University, Nathan, QLD, Australia; ^3^Institute of Glycomics, Griffith University, Gold Coast, QLD, Australia

**Keywords:** protozoan parasites, Th1 cells, IL-10, IL-27, CD4^+^ T cells, immune regulation

## Abstract

CD4^+^ T cells are critical for defense against protozoan parasites. Intracellular protozoan parasite infections generally require the development of a Th1 cell response, characterized by the production of IFNγ and TNF that are critical for the generation of microbicidal molecules by phagocytes, as well as the expression of cytokines and cell surface molecules needed to generate cytolytic CD8^+^ T cells that can recognize and kill infected host cells. Over the past 25 years, much has been learnt about the molecular and cellular components necessary for the generation of Th1 cell responses, and it has become clear that these responses need to be tightly controlled to prevent disease. However, our understanding of the immunoregulatory mechanisms activated during infection is still not complete. Furthermore, it is apparent that although these mechanisms are critical to prevent inflammation, they can also promote parasite persistence and development of disease. Here, we review how CD4^+^ T cells are controlled during protozoan infections and how these regulatory mechanisms can influence parasite growth and disease outcome.

## Introduction

Mammalian immune systems have evolved to recognize and control pathogens. This is achieved by the coordinated actions of innate and adaptive immune mechanisms [reviewed in Ref. (1, 2)]. CD4^+^ T cells play key roles in coordinating immune responses by producing molecules critical for the production of high affinity antibodies by B cells and promoting the production of mucous and tissue repair mechanisms. They also help to fully activate CD8^+^ T cells so they can kill infected and transformed cells, and assist innate immune cells to recognize and control pathogens and tumors. CD4^+^ T cells play critical roles in both the generation of anti-parasitic immunity and immune surveillance during concomitant immunity, which is associated with many parasitic infections (3).

## Regulation of T Cell Responses

The help provided by CD4^+^ T cells for various immune activities includes the production of potent pro-inflammatory cytokines such as TNF, IFNγ, and IL-17, and as such, CD4^+^ T cell responses need to be tightly regulated so they themselves do not cause tissue damage. The pathogenesis of autoimmune diseases often involves aberrant CD4^+^ T cell responses in tissue sites such as the central nervous system, pancreas, and brain. Therefore, mammals have evolved multiple ways to control the pathogenic potential of CD4^+^ T cells [reviewed in Ref. (4)]. These include indoleamine 2,3-dioxygenase (IDO)-catalyzed tryptophan metabolism by phagocytic cells (5), leading to immune cell stress and activation of the general controlled non-repressed 2 (GCN2) kinase pathway (6) and/or cytotoxic and regulatory effects on T cells caused by the catabolites from the associated kynurenine metabolism pathway (7). In addition, the production of regulatory cytokines, such as IL-10 and TGFβ, by innate immune cells in response to pathogen-derived molecules can suppress both developing and established T cell responses (8–10), as can IL-10 produced by certain B cell subsets (11). Dendritic cells (DCs) can be an important source of regulatory cytokines in experimental models of leishmaniasis and malaria. In addition, over the course of these infections, DCs reduce levels of CD11c, increase expression of CD45RB, and promote the generation of T cell IL-10 production (12–14). Thus, the development of regulatory DC subsets that have a major influence on T cell responses is a feature of established protozoan infections. More recently, specialized monocytes and macrophage subsets have been identified that can modulate localized T cell responses during protozoan infections [reviewed in Ref. (15)]. Classically activated (M1) macrophages produce pro-inflammatory molecules, such as TNF and l-arginine-dependent nitric oxide, while alternatively activated (M2) macrophages use arginase 1 to convert l-arginine to polyamines, which along with production of IL-10 and TGFβ, enable this cell subset to suppress inflammation [reviewed in Ref. (16)]. Inflammatory monocytes have been reported to promote Th1 cell activity in mice infected with *Leishmania major* (17), *L. donovani* (18), and *Trypanasoma brucei* (19, 20), but with pathological consequences in the latter model that were reversed by administration of IL-10 (20). In contrast, the products from M2 macrophages suppressed lesional CD4^+^ T cell proliferation and IFNγ production in mice infected with *L. major* (21), while *T. gondii* can actively promote the arginase 1 pathway in macrophages to enhance pathogen survival (22). Thus, macrophages play important roles in conditioning local tissue environments and determining the direction and effectiveness of T cell responses during protozoan infections. However, regulatory mechanisms increasingly recognized as being paramount for preventing T cell-mediated disease, and therefore, the main subject of this review, involve specialized sub-populations of CD4^+^ T cells themselves capable of inhibiting immune responses and suppressing inflammation.

## Regulatory T Cells

Regulatory T cells can be broadly divided into two types. First, natural regulatory T (Treg) cells are CD4^+^ T cells produced in the thymus and express the transcription factor FoxP3 that is critical for their suppressive functions (23, 24). Second, inducible regulatory T cells emerge from the thymus as conventional T cells, but develop regulatory functions in the periphery following exposure to appropriate inflammatory stimulation. These include IL-10-producing Th1 (Tr1) cells (25), TGFβ-producing CD4^+^ T (Th3) cells (26), and conventional CD4^+^ T cells that have converted to FoxP3-positive cells in peripheral tissues (27). Under homeostatic conditions, Treg cells limit potentially self-reactive T cell responses, thus preventing autoimmunity (23). However, they can also impair effective pathogen clearance, while trying to prevent immune-mediated tissue damage during infection. The molecular mechanisms by which Treg cells perform these functions are incompletely understood, but involve production of cytokines such as IL-10, TGFβ, and IL-35, the expression of the negative regulatory molecule cytotoxic T lymphocyte-associated antigen 4 (CTLA-4) and the generation of adenosine and cyclic AMP [reviewed in Ref. (28)]. In addition, their expression of high affinity IL-2 receptor allows them to deprive conventional T cells of this critical growth factor and thereby induce them to undergo apoptosis (29). Thus, Treg cells may act directly upon conventional T cells or via accessory cells such as antigen presenting cells (APCs) to limit T cell activity. An emerging paradigm is that Treg cells adapt to particular inflammatory conditions in order to regulate specific CD4^+^ T cell responses by the generation and use of shared transcription factors to mimic certain aspects of T cell behavior, such as tissue homing, survival, and cytokine production. For example, STAT3, T-bet, IRF-4, and Bcl-6 are required for Th17, Th1, Th2, and follicular helper T (Tfh) cell differentiation, respectively, as well as by the Treg cells that control the actions of these specific CD4^+^ T cell subsets (30–34). For example, in mice orally infected with *T. gondii*, IL-27 promotes the expression of CXCR3 on Treg cells that enables these cells to regulate Th1 cell-mediated immunity, as well as prevent infection-induced pathology at mucosal sites (35). However, Treg cells can block the generation of effective parasite-specific T cell responses in specific tissues. For example, Treg cell depletion with anti-CD25 mAb in mice infected with *L. major* dramatically enhanced anti-parasitic immunity (36), while adoptive transfer of antigen-specific Treg cells in the same model promoted parasite growth (37). In addition, depletion of Treg cells with an anti-CD25 mAb protects mice from lethal *Plasmodium yoelii* infection by enabling the generation of a potent anti-parasitic T cell response (38). Similarly, the removal of Treg cells from peripheral blood mononuclear cells isolated from humans infected with *P. falciparum* enhanced T cell proliferation and CD4^+^ T cell IFNγ production in response to stimulation with parasite antigens (39). However, the importance of Treg cells in several protozoan infections has been questioned because of the potential “off-target” effects of the anti-CD25 mAbs used in many studies (40). For example, several groups reported significant changes in immune responses and disease outcome in mice infected with *P. berghei* ANKA (41–43), but subsequent experiments in this model, where Treg cells could be specifically depleted with diphtheria toxin via cell-specific expression of a simian diphtheria toxin receptor (44), showed little impact of Treg cells on disease outcome and associated T cell responses (45, 46). Thus, the roles of Treg cells in protozoan infections will require further studies before their impact on anti-parasitic immune responses can be fully appreciated.

The secretion of IL-10 by conventional CD4^+^ T cells can potently suppress inflammation and tissue damage (47, 48). Initially, IL-10 production was identified in Th2 cells (49), but has since been described in Th1 (50–52) and Th17 (53) cell populations. Thus, CD4^+^ T cell-derived IL-10 production is emerging as an important mechanism of auto-regulation, whereby IL-10 can both directly suppress T cell activities, as well as upstream activation pathways initiated by APCs [reviewed in Ref. (48)]. These IL-10-producing Th1 cells were identified in mice infected with *T. gondii* (54) and *L. major* (55). In the *T. gondii* infection model, these cells did not impact upon control of parasite growth, but were critical for limiting pathology (54, 56), while in mice infected with *L. major*, IL-10-producing Th1 cells promoted the establishment and maintenance of chronic infection (55). Similar observations have also been made in mouse models of *Plasmodium* infection (57, 58), *T. cruzi* (59, 60), and *T. brucei* (61) infections. Importantly, these IL-10-producing Th1 cells have been identified in humans with visceral leishmaniasis caused by *L. donovani* (62) and African children with *P. falciparum* malaria (63, 64). Although IL-10 has been clearly shown to suppress CD4^+^ T cell activation in humans infected with *L. donovani* (65) and *P. falciparum* (63, 66), it is not yet clear how much of this activity can be attributed to the IL-10-producing Th1 cells. Significantly, the prevalence of IL-10-producing Th1 cells in Gambian children with asymptomatic malaria was greater than in children with severe disease, indicating that these cells may protect against damaging inflammation during acute malaria (67). However, antigen-specific IL-10-producing Th1 cells were found in cord blood of babies whose mothers had malaria during pregnancy (66), suggesting that these cells might be able to influence anti-parasitic immunity from very early in life. Hence, the kinetics of the emergence of IL-10-producing Th1 cells during malaria may be critical in determining the impact they have on the outcome of infection.

## The Roles of IL-10 in Protozoan Infections

IL-10 is one of the most potent regulatory cytokines produced by leukocytes in response to inflammatory signals (68). The importance of IL-10 for regulating immunity is highlighted by the observation that IL-10 deficiency or blockade causes the early development of colitis in mice (69). However, as described above, many protozoan parasites, such as those that cause toxoplasmosis, malaria, trypanosomiasis, and leishmaniasis (54, 55, 59–61, 70, 71), have evolved to exploit the functions of IL-10 to inhibit anti-microbial mechanisms and allow the establishment of chronic infection. In fact, the generation of IL-10-producing T cells following vaccination with protozoan antigen can be a robust predictor of vaccine failure (28). One proposed mechanisms for IL-10-mediated immune suppression is the promotion of T cell exhaustion. The PD-1 pathway plays an important role in T cell exhaustion during all of the chronic infections mentioned above, and there is strong evidence that IL-10 plays a key role in regulating the expression of the PD-1 ligands (PD-L1 and PD-L2) on APCs [reviewed in Ref. (72)]. Several other molecules known to be involved in promoting T cell exhaustion, such as Tim-3 and Lag3, have also been linked with IL-10 expression (73, 74), but their precise relationships are not known. IL-10 produced by macrophages can also inhibit the differentiation of surrounding cells into classically activated macrophages that are required for the production of inflammatory cytokines and metabolites required to kill many intracellular pathogens (75). It can also suppress inflammatory cytokine production by T cells and inhibits antigen presentation by APC [reviewed in Ref. (76)]. Thus, IL-10 can suppress host immune responses during infection by multiple mechanisms.

## Regulation of IL-10 Production by CD4^+^ T Cells

An important approach to understanding how IL-10 production might be modulated for therapeutic advantage or to improve vaccination is to gain a better insight into the transcriptional regulation and the signaling pathways involved in IL-10 production and establishing whether they differ between cell types and in various tissue locations. IL-27 has emerged as an important growth and differentiation factor for IL-10-producing Th1 cells (53, 77, 78). It is thought to primarily be a product of macrophages and DCs (79), and drives the production of IL-21 by CD4^+^ T cells, which in turn, acts as an autocrine growth factor for IL-10-producing Th1 cells (80, 81). IL-27 is a heterodimeric cytokine composed of IL-27p28 and EBI3 that signals via a receptor complex comprising a unique IL-27 receptor alpha chain (IL-27Rα) and gp130 (82, 83), a common receptor used by several cytokines including IL-6 (84, 85). IL-27 promotes these activities via the transcription factors STAT1 and STAT3 (53), and by inducing the expression of the transcription factors c-Maf (80) and aryl hydrocarbon receptor (AhR) (86), which then physically associate and transactivate the IL-10 and IL-21 gene promoters (80, 86, 87). Interactions between glucocorticoid-induced TNFR-related (GITR) protein and GITR ligand can also stimulate IL-27 production (88), which can induce expression of inducible T cell costimulator (ICOS) on IL-10-producing Th1 cells to enhance IL-27-mediated expansion of these cells (80). Interestingly, IL-27p28 can also function as a natural antagonist of gp130-mediated cytokine signaling, and thereby inhibit IL-6-mediated inflammatory pathways (89). The importance of IL-27 for the generation of IL-10-producing Th1 cells has now been reported in mouse models of malaria (58, 90), leishamaniasis (91), and toxoplasma (53), although, surprisingly, the generation of these cells was independent of IL-21 in mice infected with *P. chabaudi* (58). It should also be noted that IL-27 has IL-10-independent regulatory functions in mice infected with *P. berghei* NK65 (90), thus emphasizing the complexity of IL-27-mediated immune regulation during protozoan infections. IL-27 produced by CD14 positive monocytes was also reported to be associated increased numbers of IL-10-producing Th1 cells in blood from visceral leishmaniasis patients (92). Thus, there is substantial evidence for IL-27 being a critical factor in the generation of IL-10-producing Th1 cells during protozoan infections.

**Figure 1:**
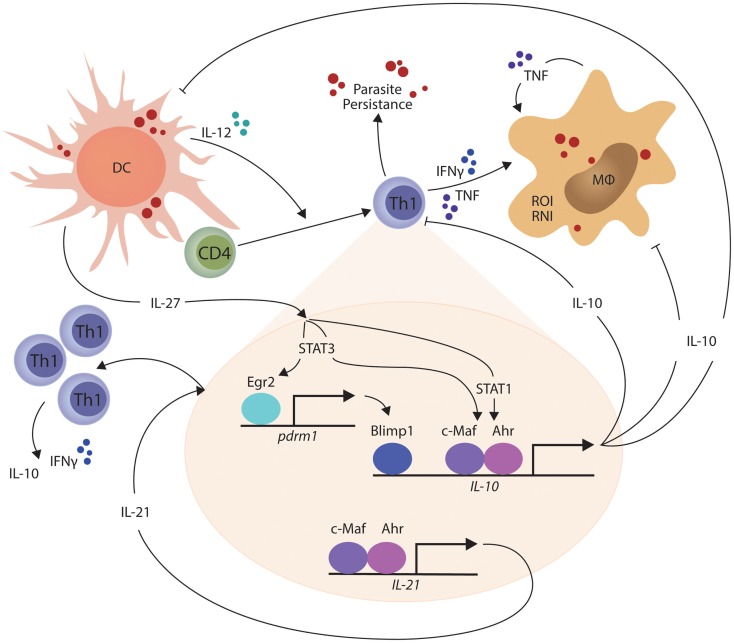
**IL-27-mediated generation of IL-10-producing Th1 cells is shown**. In the presence of persistent parasite antigen exposure, IL-27 from macrophages and dendritic cells (DCs) stimulates STAT1 and STAT3-dependent transcription of c-Maf and aryl hydrocarbon receptor (AhR) in Th1 cells, which then physically associate and bind the IL-10 and IL-21 gene promoters to drive gene transcription. IL-27 and T cell receptor signaling also combine to promote the expression of the transcription factor Erg2, which is critical for the induction of Blimp1 and generation of IL-10-producing Th1 cells in a STAT3-dependent manner. IL-21 acts as an autocrine growth factor for IL-10-producing Th1 cells, while the IL-10 produced by these cells can suppress the inflammatory functions of Th1 cells and phagocytes, as well as the antigen presenting capacity of DCs, macrophages (MØ), and monocytes (MO). The small red circles represent protozoan parasites and associated antigens.

In other studies, the transcriptional repressor B lymphocyte-induced maturation protein 1 (Blimp1; encoded by the *Prdm1* gene) was found to be expressed by a subset of Treg cells and played an essential role in their production of IL-10 (93). Recently, Blimp1 was implicated in IL-10 production by Th1 cells (94), and shown to be important for the generation of these cells in mice infected with *T. gondii* (95). IL-27 and T cell receptor signaling were found to promote the expression of the transcription factor Erg2, which was required for Lag3 expression and production of IL-10 by conventional CD4^+^ T cells (96). Subsequently, IL-27-dependent Egr2 expression was reported to be critical for the induction of Blimp1 and generation of IL-10-producing Th1 cells (94). Interestingly, only STAT3-deficiency impacted upon IL-27-dependent Egr2 expression, while both STAT1 and STAT3 were required for IL-10 production by Th1 cells. Thus, a model for IL-10 production by Th1 cells is emerging (Figure 1). Furthermore, cellular pathways such as the Notch-Jagged axis in plasmacytoid DCs promote CD4^+^ T cell IL-10 production (97), but their roles in protozoan infections have yet to be investigated. Thus, there are still many gaps to be filled, and importantly, we need to clearly define the signaling and transcriptional pathways that are activated during protozoan infections. In particular, there is a clear gap in our knowledge regarding differences in the regulation and maintenance of IL-10 production by Th1 cells in secondary lymphoid organs and peripheral tissue sites. This information is important if we want to target these regulators to selectively modulate IL-10 activity during parasitic disease. In the broader context of immune regulation, we need to establish whether the IL-10-producing Th1 cells are a distinct T cell subset capable of dynamic and sustained regulatory function or whether they represent exhausted T cells, as suggested by their expression of molecules such as PD-1 and Lag3. In the former, we can develop ways to manipulate them for therapeutic advantage (for example in inflammatory diseases) or transiently block their function, as might be required for effective vaccination. However, if they represent a terminally differentiated state, then different approaches may have to be devised to either promote or inhibit their development.

## Other Mechanisms of Th1 Cell Regulation during Protozoan Infections

**Figure 2:**
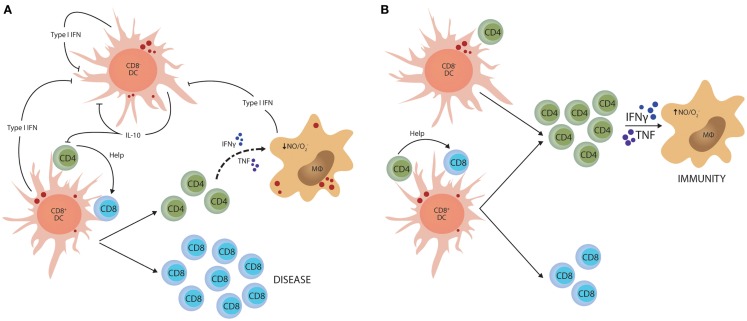
**Type I IFN-mediated suppression of Th1 cell activation**. **(A)** Parasite molecules stimulate type I IFN production by different dendritic cell (DC) subsets and macrophages (MØ). This family of cytokines feedback on these innate immune cells and suppress their capacity to activate CD4^+^ T cells. In experimental malaria caused by *Plasmodium berghei* ANKA, this effect is primarily directed toward the CD8^−^ DC subset and stimulates IL-10 production. **(B)** In this model, blockade of type I IFN signaling dramatically enhances the generation of anti-parasitic CD4^+^ T cell responses that can protect mice from CD8^+^ T cell-mediated severe disease. The small red circles represent protozoan parasites and associated antigens.

Although IL-10 is a potent regulator of Th1 cell responses, there is likely to be multiple mechanisms to control such potentially damaging inflammatory responses. Type I IFNs have recently emerged as import immune regulators during parasitic infections. They are produced by most cell types and play critical roles in anti-viral immunity (98, 99), but several studies have identified this family of cytokines as important determinants of disease outcome in protozoan infection. However, these effects depend on the virulence of the parasite and the stage of infection. For example, in mice lacking type I IFN receptor, early control of *T. brucei* was impaired, but it appeared that IFNγ production later in infection was suppressed by type I IFN signaling pathways (100). In contrast, early control of a high dose *T. cruzi* infection was enhanced in type I IFN receptor-deficient mice and this was associated with increased IFNγ production, but not when a lower parasite dose was used (101). In liver stage *P. berghei* ANKA infection, parasite RNA triggers a type I IFN transcriptional program in hepatocytes that enhances innate immune responses in hepatic myeloid cells to control liver parasite load (102). In contrast, in mice infected with *P. berghei* ANKA blood-stage parasites, type I IFNs promoted susceptibility to severe disease (103, 104) by suppressing Th1 cell development (103) indirectly through inhibition of CD8^−^ DC function (105). Interestingly, this latter effect of type I IFN on DC function was also associated with reduced IL-10 mRNA accumulation in CD8^−^ DCs that lacked type I IFN receptor, potentially linking infection-induced IL-10 production with Th1 regulation once again (Figure 2).

TGFβ has also emerged as an important regulatory cytokine controlling Th1 responses during protozoan infections [reviewed in Ref. (106)]. In mice, susceptibility to lethal *P. berghei* ANKA infection correlated with reduced TGFβ levels (107), while high levels of TGFβ in malaria patients was associated with increased parasite growth. In mice infected with *T. gondii*, TGFβ produced by gut intraepithelial CD8^+^ T cells was critical for controlling inflammation and gut pathology (108), thus supporting a key role for this cytokine in regulating inflammation during protozoan infections. In both malaria and toxoplasma, it is likely that TGFβ acts by suppressing T cell activation and promoting Treg cell functions (109). However, this is yet to be formally demonstrated and there still remains much to learn about the functions of TGFβ during infection.

Both lipoxin A4 and glucocorticoids have also been identified as important regulators of Th1 cell responses in mice infected with *T. gondii* (110–112). Lipoxin A4 is an eicosanoid mediator capable of suppressing DC IL-12 production in response to parasite antigen *in vitro* (110) or during *T. gondii* infection in mice (111). In this latter study, infection of mice lacking lipoxin A4 resulted in a fatal, parasite-induced inflammation (characterized by a potent Th1 cell response), but reduced parasite loads. Importantly, results from this and previous studies (113), suggest that IL-10 was critical for regulating inflammation during the acute stage of infection, while lipoxin A4 was important for immune regulation during chronic infection (111). A novel, IL-10-independent pathway of immune regulation was also recently identified in this infection model, whereby glucocorticoids produced by the hypothalamic–pituitary–adrenal axis during *T. gondii* infection act directly on CD4^+^ T cells to prevent Th1 cell hyperresponsiveness and resulting pathology (112). Given the critical roles for both IL-10-dependent and IL-10-independent pathways in preventing inflammatory diseases associated with protozoan infections, temporal and/or transient blockade of one or the other pathways may be a viable way to enable sufficient pro-inflammatory immunity to control parasite growth, but also leave enough regulatory machinery in place to prevent disease.

## Concluding Remarks

There are currently no vaccines to protect against or treat diseases caused by protozoan parasites. It has proven extremely difficult to generate robust and long-lasting CD4^+^ T cell responses against the responsible pathogens (3). An important impediment for generating sufficient immunity against these pathogens could, in some cases, be the accompanying regulatory immune response that aims to limit inflammation. Treg cell depletion can dramatically improve candidate malaria vaccine efficacy (114, 115), although as mentioned above, these studies must be interpreted with caution because of the use of anti-CD25 mAb for Treg cell modulation. Nevertheless, the blockade of IL-10 produced by antigen-specific Th1 cells improved anti-parasitic immunity generated by a candidate vaccine directed against *L. major* (116), while studies on *T. gondii* indicated that induction of IL-10-producing Th1 cells following vaccination caused a lethal infection upon secondary exposure to the parasite (117). Although the depletion of Treg cells and/or IL-10-producing Th1 cells is not a viable option for improving vaccine efficacy given the critical roles of these cells in preventing immune-mediated disease, a much better understanding about how regulatory immune responses can be locally and temporarily modulated to enhance vaccine-induced immune responses may be of significant benefit.

The regulation of Th1 cell responses during protozoan infections is clearly important to ensure both sufficient generation of inflammatory mediators to control parasite growth, as well as to prevent excessive production of these molecules in sensitive tissue sites. IL-10 has emerged as an important regulator of these responses, both produced in a highly regulated manner by Th1 cells themselves, as well as parasite-activated innate immune cells. However, IL-10 is not alone in this activity, and alternative mechanisms of Th1 cell regulation have been identified. Our challenge remains to fully define these mechanisms of Th1 cell regulation and to use this knowledge to improve therapeutic options and vaccine efficacy. Research in protozoan infections of both mice and humans is ideally placed to identify broad mechanisms of immune regulation that are relevant not only to parasitic infections but also for autoimmune and physiological diseases, as well as cancer.

## Author Contributions

Christian R. Engwerda, Susanna S. Ng, and Patrick T. Bunn all contributed to the planning and research in this paper. Susanna S. Ng conceived and produced both figures, while Christian R. Engwerda and Patrick T. Bunn wrote the paper.

## Conflict of Interest Statement

The authors declare that the research was conducted in the absence of any commercial or financial relationships that could be construed as a potential conflict of interest.
